# Giant Intraperitoneal Multiloculated Pseudocyst in a Male

**DOI:** 10.1155/2016/4974509

**Published:** 2016-02-24

**Authors:** Jacqueline Oxenberg

**Affiliations:** Department of Surgical Oncology, Geisinger Wyoming Valley, Wilkes-Barre, PA 18711, USA

## Abstract

Intraperitoneal pseudocysts are rare and may be difficult to differentiate from other malignant neoplasms. Reports of occurrences are mainly associated with long-term intraperitoneal catheter use, intraperitoneal catheter infections, or major pelvic surgery in females, although there are few reported incidences without prior trauma. We present a case of a male patient found to have a 19 × 15 × 9 cm intraperitoneal pseudocyst with other multiloculated areas and a history of a right inguinal hernia repair. After a thorough review of the literature, this is the first multiloculated nonpancreatic pseudocyst reported in a male patient.

## 1. Introduction

Intraperitoneal pseudocysts are rarely reported and are mainly secondary to inflammatory insults such as ventriculoperitoneal shunts, catheter infections, or even major pelvic surgery in females [1–8]. Only few reports of intraperitoneal pseudocysts without iatrogenic causes exist and none are multiloculated [9–11]. While most are benign, differentiation between a malignant process can be difficult. We present a case of a male patient found to have a giant multiloculated intraperitoneal pseudocyst.

## 2. Case Report

**Figure 1:**
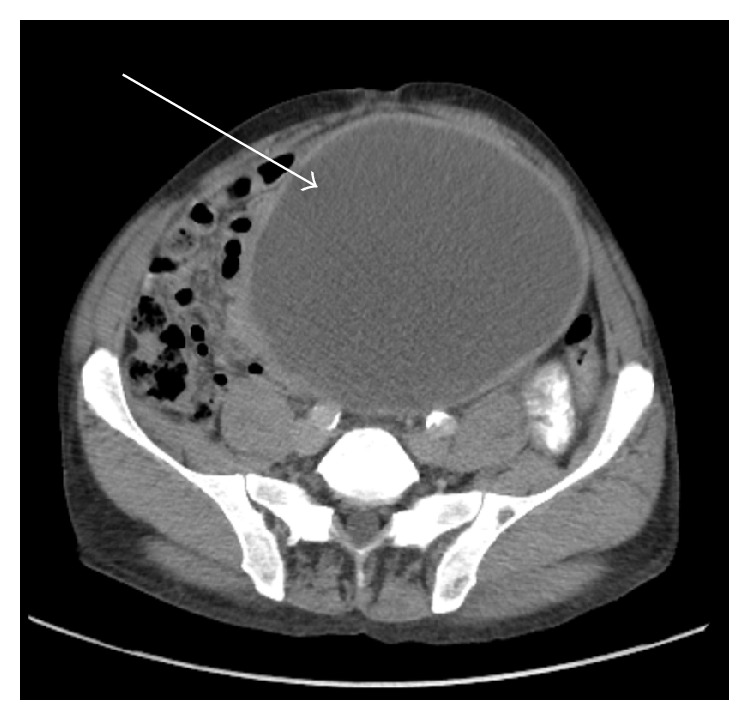
Large intraperitoneal cystic mass measuring 17.2 × 14.6 × 17.3 cm with displacement of bowel.

**Figure 2 fig2:**
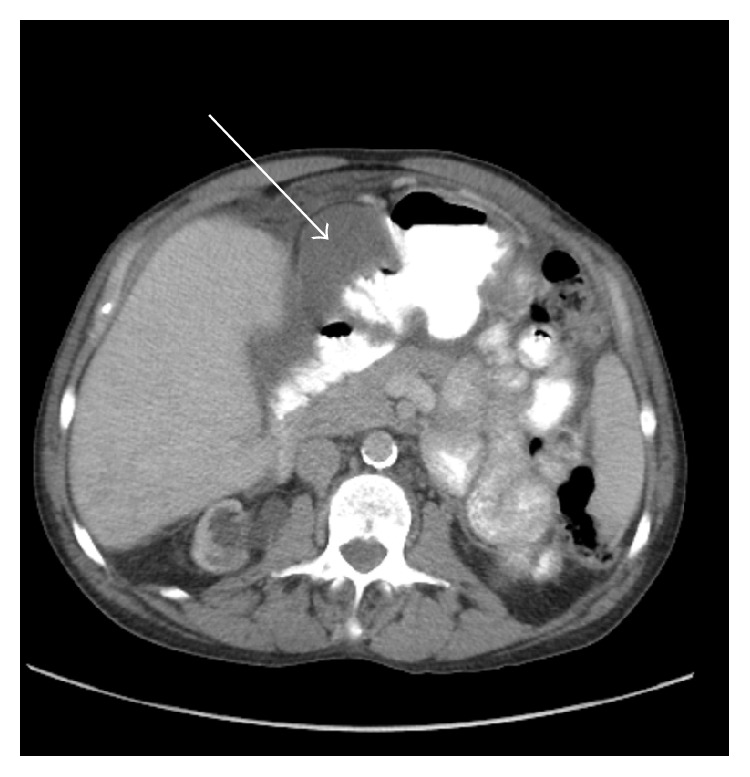
Second separate loculated area measuring 3.7 × 10.1 × 3.8 cm measuring 24 Hounsfield units.

**Figure 3:**
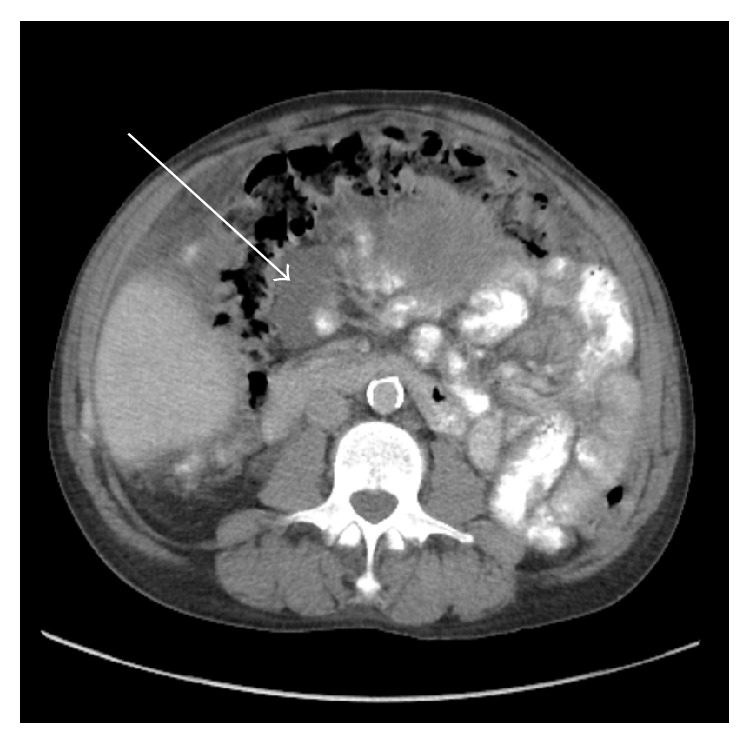
Smaller loculated fluid collection within the right midabdomen between the mesocolon of the proximal transverse colon/hepatic flexure and small intestine.

**Figure 4:**
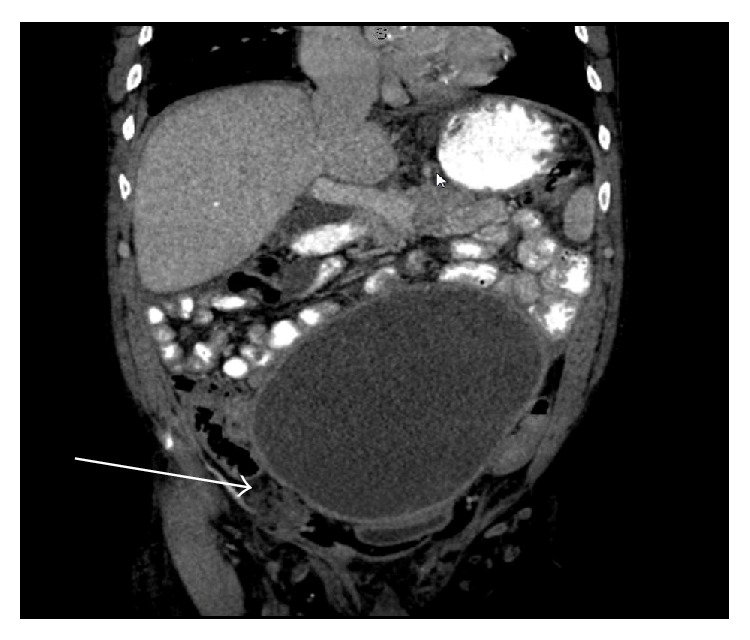
Site of prior right inguinal hernia repair adjacent to the pseudocyst.

A 58-year-old male presented to his primary care physician with central abdominal pain and distension. His past medical history was significant for hypertension, hyperlipidemia, and end-stage renal disease secondary to hypertensive kidney disease requiring hemodialysis. This was performed via arteriovenous fistula starting 8 years prior to being seen. He denied any history of peritoneal dialysis and his only abdominal surgery was a right inguinal hernia repair approximately 4 years before. Unfortunately, the operative report was unavailable to us and he was unsure if mesh was used. CT of the abdomen and pelvis with oral and intravenous contrast showed a 17.2 × 14.6 × 17.3 cm large pelvic cystic tumor with separate loculated areas in the right upper abdomen adjacent to the gastric antrum. A smaller fluid collection in the right midabdomen between the mesocolon of the proximal transverse colon/hepatic flexure and small intestine, ascites, and thickened omentum were also seen (Figures 1 [Fig fig2]–3). Hounsfield units for the cystic regions were 24. There was also a loculated right pleural effusion. The appendix could not be visualized (Figure 1). The prior right inguinal hernia repair with possible mesh could be seen adjacent to the pseudocyst (Figure 4). He presented to the surgical oncology clinic for further workup. At the time, he only complained of abdominal pain and distension without any other associated symptoms. His baseline creatinine was 5.3 mg/dL (normal 0.6–1.3 mg/dL). His white blood cell count was 4.4 k/uL (normal 4–10.8 K/uL), albumin was 3.6 g/dL (normal 3.8–5.0), and carcinoembryonic antigen (CEA) was 3.6 (normal < 3.5). The patient was a nonsmoker and denied weight loss.

He underwent a bronchoscopy that showed extrinsic compression from the loculated effusion. Washings showed atypical cells but no malignancy was identified. His information was reviewed and discussed in our gastroenterology multidisciplinary conference and a decision was made to proceed with surgical exploration and resection of the large intra-abdominal tumor. Given the possibility for mucinous tumor, surgical debulking as well as potential intraperitoneal chemotherapy was also discussed.

**Figure 5:**
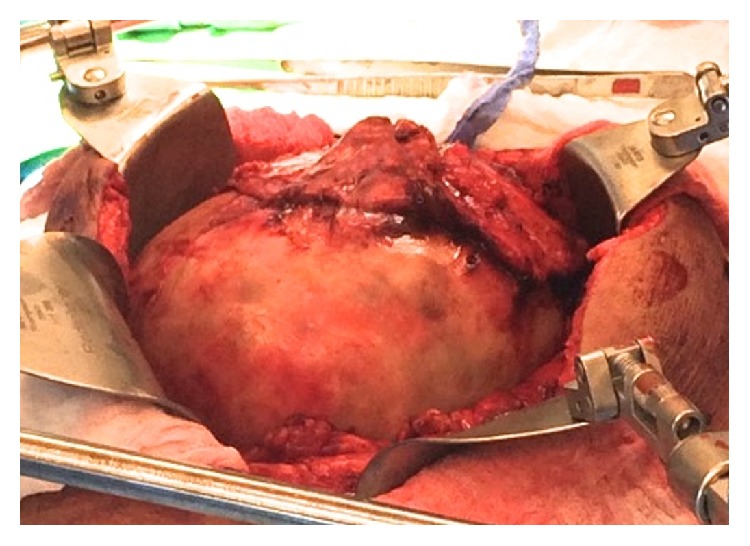
Large intraperitoneal mass exposed intraoperatively with adherent omentum.

During surgery, the tumor was found to be adherent to the omentum and anterior abdominal wall and was unable to be separated requiring resection of a portion of peritoneum. This was suspected to be close to the region of the hernia repair, although it was difficult to discern if scar tissue or tumor caused the inability to separate the tumor from the peritoneum. No mesh was encountered in this space. The mass was also adherent to the right colon but was able to be separated with careful dissection. It was not attached to the appendix, but this was removed given the original concern for mucinous ascites. The tumor appeared encapsulated with a tan-yellow capsule and multiple thick adhesions were encountered (Figure 5). Multiple nonencapsulated pockets of ascites were also found throughout the abdomen consistent with findings on the CT. The pocket in the right midabdomen was removed intactly and also appeared as a tan-yellow cystic structure. His surgery included an exploratory laparotomy, excision of the intra-abdominal tumor with appendectomy, greater and lesser omentectomy, excision of the falciform ligament, and peritoneal stripping at the area of attachment to the abdominal wall.

**Figure 6:**
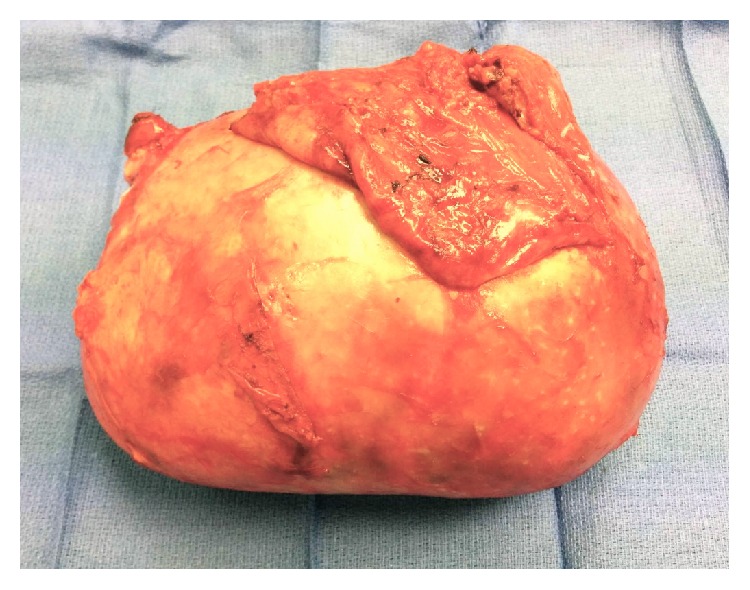
Intact large cystic mass measuring 19 × 15 × 9 cm with a thick tan-yellow capsule.

**Figure 7:**
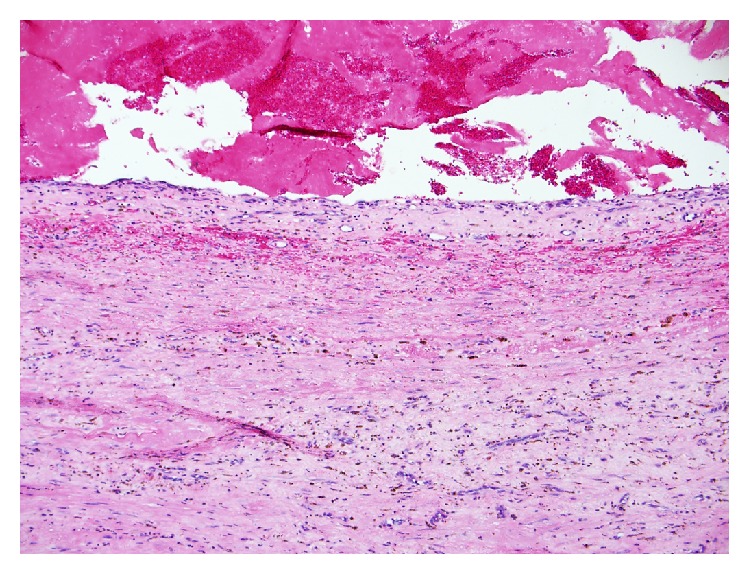
Micrograph shows cystic wall composed of fibrous tissue. There are angiogenesis, mild chronic inflammation, and hemosiderin deposits. Hemorrhagic material is seen loosely attached to the luminal surface. H&E section (100x).

**Figure 8:**
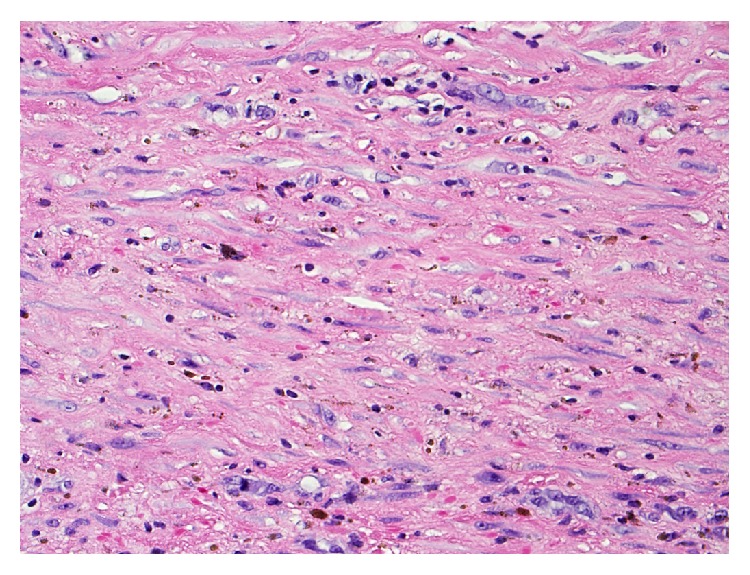
High power view of the cystic wall shows reactive fibrosis. The fibroblast contains spindle nuclei with small nucleoli set against a dense collagenous matrix. There are scattered foamy macrophages, lymphocytes, and hemosiderin pigments. H&E section (400x).

Final pathology revealed an intact large cystic mass measuring 19 × 15 × 9 cm (Figure 6). Grossly, the lesion was tan-yellow and smooth and had a dull external surface with thin fibrous adhesions. Upon opening the cyst, a large amount of hemorrhagic fluid was seen. The inner wall was covered by a friable slightly necrotic and hemorrhagic appearing exudate. Microscopic examination showed the cystic wall was composed of fibrous tissue with mild chronic inflammation and hemosiderin deposits (Figure 7). The luminal surface was lined by granulation tissue and fibrin. Recent hemorrhagic material was also noted attached to the luminal surface. The fibrous wall measured up to 0.8 cm thick. Microscopic evaluation revealed organizing hemorrhage with surrounding dense fibrous cyst wall and stromal reactive changes. There was no evidence of neoplasm within the cyst wall, nor was any concerning neoplasm found within the appendix or omentum (Figure 8). Pathology at our institution as well as at the Mayo Clinic confirmed the final diagnosis to be a giant pseudocyst. The appendix, omentum, smaller cystic structure, and falciform were all without malignancy. The smaller cyst showed fibroadipose tissue with chronic inflammation and hemorrhage.

The patient recovered well postoperatively without complication and was discharged home on postoperative day 6. Most recent imaging performed approximately 3 months later showed no intraperitoneal recurrence, but he required a right thoracentesis for a recurrent pleural effusion. CEA was not rechecked given that no carcinoma was found.

## 3. Discussion

We present a case of a giant multiloculated intraperitoneal pseudocyst in a male patient. Few prior reported cases without a history of surgical intervention have been found in the literature [9–11]. Reports of nonpancreatic pseudocysts secondary to surgery included major pelvic surgery in premenopausal females where the ovaries were demonstrated within the cysts [5, 8]. Other case reports or small series with pseudocysts secondary to surgery include ventriculoperitoneal shunts and intraperitoneal dialysis catheters, especially after infection [5, 8, 12]. Given that the peritoneum can be disrupted or violated during a hernia repair, it is possible that this may have been the inciting event, although only uniloculated nonpancreatic pseudocysts have been only reported without iatrogenic causes [9–11]. While his chronic renal failure may be a mitigating factor suggesting a systemic component, especially given his pleural effusions, pseudocyst formation without peritoneal dialysis catheter placement has not been reported. The mechanism of a traumatic pseudocyst has been described as secondary to a hematoma or abscess that failed to resolve [13]. Although traumatic injury secondary to hernia repair is likely, given the rare presentation and multiloculation, other possibilities need to be considered.

de Perrot and colleagues proposed a classification of mesenteric cysts that included (1) cysts of lymphatic origin; (2) cysts of mesothelial origin; (3) cysts of enteric origin; (4) cysts of urogenital origin; (5) mature cystic teratoma; and (6) pseudocysts [14]. Older classifications by Beahrs et al. and Ros et al. also included cysts as traumatic and nonpancreatic pseudocysts, respectively [15]. Given the clinical findings and pathological characteristics, this was not a cyst of lymphatic, mesothelial (mesothelial cells were not seen), enteric, or urogenital origin. It also did not have features of a teratoma. It is also possible this patient may have had a mesenteric cyst. The classic description of a mesenteric cyst includes a single layer of columnar or cuboidal epithelial cells, but this layer may become destroyed as a result of pressure from the cyst fluid [12, 16]. The definition of a pseudocyst is a cyst without epithelial cells [17]. No columnar or cuboidal cells were seen within the largest cyst, but rather hemorrhagic material was seen. In some series, mesenteric cysts are commonly located in the small bowel mesentery, mesocolon, or even the retroperitoneum, similar to our patient [12, 18].

The largest series of intraperitoneal cysts included 16 patients with mesenteric cysts reported over 14 years [12]. This series included 44% males with abdominal pain and mass as the most common symptoms, similar to our patient, but synchronous other cystic structures were not described [12]. Mesenteric cysts may be uni- or multiloculated, where even an incidence of a patient who was thought to have ascites prior to diagnosis was found [12]. Since rupture of a mesenteric cyst is possible, especially since hemorrhage was seen on pathology, this may explain the other cystic structures seen on imaging or intraoperatively. This may also explain the recurrent pleural effusions, or hydrothorax. Although reported incidences were seen with benign serous ovarian tumors, they were also associated with ascites and hydrothorax, where a similar mechanism can be translated to our patient [19].

The clinical significance of these tumors should not be underestimated. Their large size can result in a wide range of symptoms and they may be difficult to differentiate from a neoplastic process, especially when multiloculated areas are found. Our patient only complained of distension and abdominal pain; however, they can cause a wide variety of symptoms including constipation, bowel obstruction, or even an acute abdomen secondary to infection, bleeding, rupture, volvulus, or even bowel ischemia [12, 17]. Additionally, these tumors may be difficult to differentiate from other neoplastic lesions such as a cystic lymphangioma, mucinous cystadenoma, epidermoid cyst, cystic teratoma, cystic mesothelioma, and cystic degeneration of solid tumors [13]. With a malignancy rate of 3–19%, surgical resection is often recommended for diagnosis and treatment [12]. With multiple loculated areas seen on imaging separate from the large single cystic mass, inability to visualize the appendix, and slightly elevated CEA, a mucinous neoplasm must be included in the differential diagnoses. Areas of focal loculation with low Hounsfield units can be difficult to differentiate from pseudomyxoma peritonei [20]. Normal ascites may have a low-density image (0 Hounsfield units), whereas mucinous ascites has a significantly higher density (5–20 Hounsfield units) [21]. In the CT images demonstrated, the cystic contents or areas concerning for ascites were measured at 24 Hounsfield units, originally raising concern for mucinous ascites. Cyst aspiration was therefore not felt necessary given that the diagnosis of underlying carcinoma often requires cyst wall resection or appendectomy. Unlike other reported cases of suspected mesenteric cysts or pseudocysts, surgical exploration and resection were needed to differentiate this benign etiology from a potential neoplastic process.
